# Application of Oral Fluid Assays in Support of Mumps, Rubella and Varicella Control Programs

**DOI:** 10.3390/vaccines3040988

**Published:** 2015-12-09

**Authors:** Peter A. C. Maple

**Affiliations:** East Yorkshire Microbiology, Innovation Centre, York Science Park, York YO10 5DG, UK; E-Mail: eastyorksmicrobiol@gmail.com; Tel.: +44-0-1904-435100; Fax: +44-0-1904-435135

**Keywords:** mumps, rubella, varicella, immunization, oral fluid, antibody capture assays, time-resolved fluorescence immunoassay, genotyping, chickenpox history

## Abstract

Detection of specific viral antibody or nucleic acid produced by infection or immunization, using oral fluid samples, offers increased potential for wider population uptake compared to blood sampling. This methodology is well established for the control of HIV and measles infections, but can also be applied to the control of other vaccine preventable infections, and this review describes the application of oral fluid assays in support of mumps, rubella and varicella national immunization programs. In England and Wales individuals with suspected mumps or rubella, based on clinical presentation, can have an oral fluid swab sample taken for case confirmation. Universal varicella immunization of children has led to a drastic reduction of chickenpox in those countries where it is used; however, in England and Wales such a policy has not been instigated. Consequently, in England and Wales most children have had chickenpox by age 10 years; however, small, but significant, numbers of adults remain susceptible. Targeted varicella zoster virus (VZV) immunization of susceptible adolescents offers the potential to reduce the pool of susceptible adults and oral fluid determination of VZV immunity in adolescents is a potential means of identifying susceptible individuals in need of VZV vaccination. The main application of oral fluid testing is in those circumstances where blood sampling is deemed not necessary, or is undesirable, and when the documented sensitivity and specificity of the oral fluid assay methodology to be used is considered sufficient for the purpose intended.

## 1. Mumps: Mumps vaccination and Mumps Oral Fluid Testing

### 1.1. Mumps

Mumps is a highly infectious, generally benign disease defined by acute onset of unilateral or bilateral tender, self-limited swelling of the parotid or other salivary glands, lasting two or more days and without other apparent cause [1]. A number of complications [2,3] can result following initial infection including orchitis (20% post pubertal young men), oophoritis (5% post pubertal young women), aseptic meningitis (15%), encephalitis (1 in 6000) and pancreatitis (up to 5%). Mumps is a common cause of acquired sensorineural hearing loss [4] usually of sudden onset, unilateral and reversible but in some cases nerve damage can result in permanent and profound hearing loss. There is some debate as to whether mumps acquired during the first trimester [5] results in a higher risk of spontaneous abortion.

The causative agent of mumps, the mumps virus, belongs to the family *Paramyxoviridae*, subfamily *Paramyxovirinae*, genus *Rubulavirus*. It is a non-segmented, negative strand RNA virus comprising a helical nucleocapsid surrounded by a lipid envelope. The complete genome of mumps virus has been sequenced [6] at 15,384 nucleotides long (Genbank accession no. AF2014730) and consists of seven different transcription units [7] including nucleoprotein (NP), membrane or matrix (M), fusion (F) and haemagglutinin-neuraminidase (HN) genes organized 3'-NP-P-M-F-SH-HN-L-5'. The HN glycoprotein [8] plays a major role in cell infection by the virus as it binds to sialic acid in host cell membranes thereby bringing about viral attachment which is followed by membrane fusion [9] and release of the viral nucleocapsid into the host cell.

Humans, so far as is known, are the only natural host of the mumps virus although animal species including hamster, mouse, developing chick embryo and non-human primates can be infected [10] under laboratory conditions. The incubation period of mumps ranges between 14 and 24 days (95% cases) with a median of 18 days [11]. Epidemic transmission in humans is by droplet spread and it has been shown by virus culture [12] that in children infected with mumps virus, who subsequently developed parotitis, that virus can be recovered from saliva on the 11th to 15th day after exposure, two to six days prior to onset of clinical signs of disease and extending up to the fourth day of illness. Children with clinically non-apparent infection also shed mumps virus [12]. Using molecular techniques for detection of mumps virus following natural infection viral shedding was minimal after the first three days of symptoms [13].

Mumps is highly infectious and Philip *et al.* [14] in a unique study conducted during the late 1950s in which Eskimos residing on St. Lawrence Island first experienced a mumps outbreak showed an 88% attack rate. In the absence of mumps vaccination, reported mumps incidence in several countries of the WHO European region ranged up to >400 cases per 100,000 [15] in epidemic years and in the USA an annual incidence of approximately 2000 cases per 100,000 population has been reported [16]. Typically, in the prevaccine era there were epidemic periods every two to five years with children aged five to nine years most affected. The implementation of population based mumps vaccination changed all of this.

### 1.2. Mumps Vaccination

Mumps vaccination was first practiced during the 1940s using both live attenuated and inactivated vaccine preparations and it was shown that a significant reduction in the incidence of mumps in vaccinated *versus* control groups could be achieved and that mumps occurring in previously vaccinated individuals was of reduced severity [17]. The first of the attenuated mumps vaccines which currently underpin national immunization programs became available during the 1960s. The Jeryl Lynn vaccine strain, licensed in the USA in 1967, was developed from mumps virus isolated from a child who had developed unilateral parotitis and subsequently passaged in chick embryo amniotic cavity followed by chick embryo fibrobasts [18]. A number of other mumps virus vaccine strains have been developed and used for example, Leningrad-3, Urabe, Leningrad Zagreb and Rubini; however, there have been reports of aseptic meningitis and lack of immunogenicity for some of them [15,19].

In England and Wales, a national immunization program using one dose of a combined measles, mumps and rubella (MMR) vaccine given to children 12–15 months of age was commenced in 1988 and in 1996 a second dose was added, given at school entry [20]. Many countries have implemented MMR vaccination into their national immunization programs and in many cases a >90% reduction in mumps annual incidence has been achieved [21]. Unfortunately, despite the undeniable success of mumps immunisation using two doses of MMR there have been reports of a resurgence of mumps in highly vaccinated populations; for example in the UK, USA, Netherlands and Korea [22,23,24,25]. No single factor has been identified as the cause of these outbreaks although waning immunity in older vaccinated populations [26], incomplete vaccine coverage and differences in the immunogenicity of vaccine strains, e.g., Rubini [27] have been implicated as factors.

### 1.3. Application of Oral Fluid Testing to Mumps Control

The detection of mumps specific IgM in oral fluids using capture radioimmunoassay has been available since the early 1990s [28] and in England and Wales laboratory confirmation of mumps as a component of a national MMR surveillance program has been undertaken since 1994 [20,29]. Subsequently, mumps IgM radioimmunoassay was replaced by enzyme immunoassay [30] and detection of mumps RNA in oral fluid samples collected during the first 14 days after onset of symptoms is also possible [31]. Oral fluid sampling has a number of advantages over venipuncture, principally it is non-invasive and collection can be undertaken by unskilled staff or by the subject following a set of written instructions [32,33]. Other advantages are that oral fluid sampling is more accessible than blood sampling and there are no “sharps” to be disposed of post sampling removing the risk of contaminated needle injuries and repeated use of contaminated equipment. A number of commercial oral fluid collection devices are available [34,35] and particularly for HIV diagnosis/surveillance oral fluid testing has become a firmly established methodology [36,37]. A major advantage of oral fluid testing is that it increases sampling in difficult to access populations [38,39] and for MMR case confirmation/surveillance this is an important factor in modeling the impact of immunization programs [40].

**Figure 1:**
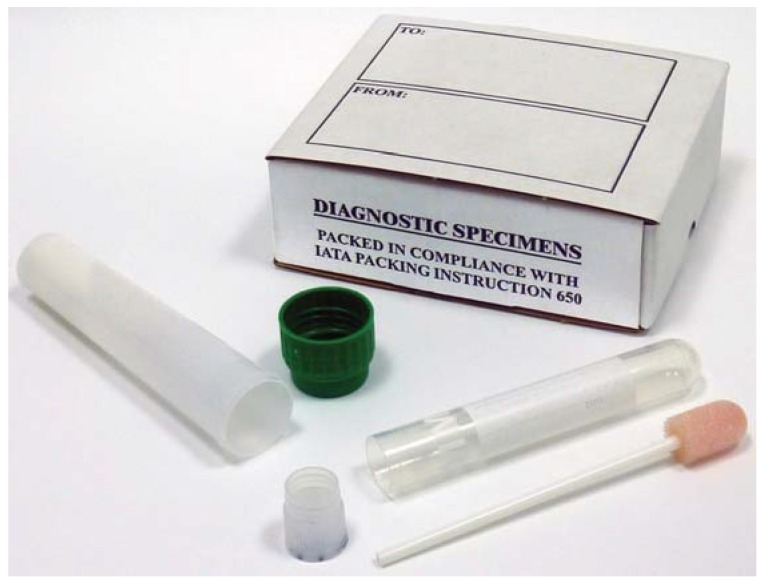
A mumps oral fluid collection kit comprising Oracol collection swab and transport packaging.

In England and Wales the Oracol oral fluid collection device is frequently used for MMR surveillance [20,34]. The device (Figure 1), either handed or posted to the test subject, comprises a sampling swab supplied with instructions for the collection of oral fluid. The subject is advised to rub the gums and teeth with the sponge head of the swab, a bit like using a toothbrush, for one to two minutes. The swab is then placed within the labeled plastic swab holder tube, capped, placed in a sealable clear plastic bag, and placed together with a completed requested form into a transport cardboard container. Finally, the transport container is placed within a pre-paid and labeled plastic envelope which can then be posted to the testing laboratory.

Testing of oral fluid for the presence of specific antibody IgG or IgM requires the use of highly sophisticated assay methodologies because the amount of specific antibody in the oral fluid sample is much less that that found within a comparable blood sample [41]. Initially, the requirement for highly sensitive detection methodologies (e.g., radioimmunoassay, time-resolved fluorescence immunoassay) limited the performance of oral fluid assays to specialized laboratories; however, commercially produced enzyme immunoassays are now available (e.g., Microimmune) and point of care tests are under development [42]. In comparison with blood based detection methodologies mumps oral fluid assays may sometimes show significantly reduced sensitivity [43]; however, in most instances sensitivity and specificity has been shown to be acceptable (Table 1).

**Table 1:** Reported performance of mumps oral fluid enzyme immunoassays (EIAs) and near patient tests.

Assay	Population Tested	Sensitivity	Specificity
IgG EIA (Clark Laboratories	157 asymptomatic subjects [44]	94.2%	93.9%
IgG capture EIA (Microimmune)	340 Norwegian conscripts [43]	80.0%	100%
IgM capture EIA (Microimmune)	137 cases of suspected mumps [30]	90.3%	97.6%
IgM near patient test	196 cases of suspected mumps [42]	79.5%	100%

Mumps oral fluid testing offers an effective means for screening the incidence of mumps and estimating levels of vaccine coverage [44,45] essential for monitoring the effectiveness of national control programs. An added advantage of oral fluid sampling is that viral RNA can be amplified [46] and genotyped [47] facilitating outbreak investigation and surveillance of mumps [47,48]. The European Region of the World Health Organization (WHO) had set a target for the reduction of the incidence of mumps to negligible levels by 2010; however, this goal remains to be achieved [49]. Mumps still proves a challenging disease to effectively control and a range of measures [50] including better ascertainment of mumps infection and vaccine effectiveness together with the potential development of new mumps vaccines will be required if mumps elimination targets are to be achieved.

## 2. Rubella: Rubella Vaccination and Rubella Oral Fluid Testing

### 2.1. Rubella

First described in 1814 and initially known as German Measles (Rötheln) until 1866 when the term “rubella” was proposed, this infection was viewed as an intermediate between measles and scarlatina [51]. By the later stages of the nineteenth century rubella was generally accepted nomenclature for some authorities; however, the descriptive German Measles was retained by others. In The Diseases of Children Medical and Surgical published 1889 [52] rubella was ascribed as an infectious fever closely resembling but distinct from measles and scarlatina and it had been observed that despite clinical similarities rubella offered no protection against subsequent measles and scarlatina and *vice versa*. Rubella frequently occurred in epidemics, had an incubation period of 14–21 days and produced confluent indistinct papules of a rose-red colour following no or an indistinct prodrome lasting one day. The potential linkage of rubella infection during pregnancy with congenital cataract and failure to thrive of the newborn combined with a high risk of congenital heart defects was first proposed by a Sydney based ophthalmologist, Sir Norman McAlister Gregg in 1941 [53]. In the cohort of patients he reviewed with the aforementioned clinical presentations he observed that most cases occurred following maternal rubella during the first two months of pregnancy and advised that exposure of the mother to infection of any kind during the entire period of pregnancy should be recorded. This truly novel finding, based on meticulous history taking, that a virus infection in humans could manifest an embryopathy was not at first universally accepted [54,55]. A limitation of Gregg’s data was that it was retrospective; however, subsequent observations of others and prospective studies [56] confirmed his hypothesis. The rate of congenital malformation following maternal rubella early in pregnancy has been shown to be very high; for example, Miller and colleagues [57] reported rubella defects in all infants infected before the 11th week of gestation.

Nowadays, with the availability of enhanced techniques for rubella virus cultivation [58], plus the availability of highly sensitive serological and molecular detection methodologies [59] combined with the ability to undertake genomic analysis [60] much more is understood about rubella and how it is spread. The rubella virus is a positive-sense, single-stranded RNA virus that belongs to the family Togaviridae and is the only member of the genus, Rubivirus. Comprising 9762 nucleotides a number of virus isolates have been whole genome sequenced [61]. The rubella virus is formed of a nucleocapsid enveloped by lipoprotein through which spikes of glycoproteins E1 and E2 protrude [62] and there are a number of non-structural proteins [63]. Both capsid and envelope glycoproteins have been shown to be immunogenic and E2 appears immunodominant [64]. Only one serotype of rubella virus appears to exist although there are a number of genotypes [65] and the virus host range is restricted to humans.

### 2.2. Rubella Vaccination

The clinical and laboratory diagnosis of rubella and congenital rubella syndrome together with the development of rubella vaccines [66,67] has been extensively reviewed elsewhere. Before the introduction of rubella vaccination into national immunization programs rubella infection occurred in endemic and epidemic waves [68] some of which were very severe. For instance, in the USA major epidemics occurred throughout the country in 1935, 1943 and 1964 against a background of periods of high incidence every six to nine years [69] During the 1964–1965 rubella epidemic in the USA there were an estimated 12,500,000 cases of rubella resulting in 159,375 cases of arthritis/arthralgia and 20,000 cases of congenital rubella syndrome [70]. The widespread use of rubella vaccination from the 1970s onwards, using differing strategies, has led to development of the goal of eradication of rubella and congenital rubella syndrome and in some countries this was achieved by the end of the last Century [71]. Recently, the World Health Assembly endorsed the target of eliminating rubella in five of the six World Health Organisation regions by 2020 to be achieved through the promotion of measles and rubella vaccination programmes combined with active surveillance for rubella and assessment of rubella immunity using a variety of methodologies including the potential use of oral fluid assays [72].

### 2.3. Application of Oral Fluid Testing to Rubella Control

Laboratory methodologies for diagnosing rubella and measuring immunity have been reviewed elsewhere [67,73] Evidence of the potential for using oral fluid samples for the detection of rubella specific IgG was first presented by Parry and colleagues [74] who tested a collection of 30 paired sera and oral fluids. Their initial findings were followed up in more detail by Perry and colleagues [28] who tested 150 oral fluid samples from patients with serologically confirmed rubella using a class G antibody capture radioimmunoassay (GACRIA). The GACRIA had 100% sensitivity for oral fluid samples collected four or more days following the onset of illness; however, the sensitivity reduced to 30%–47% for oral fluid samples collected approximately three months post onset of illness. Subsequently, the rubella GACRIA gave promising results when applied in a survey of response to rubella vaccination in Brazilian children [75]; and in a study of confirmation of notified cases of rubella in the United Kingdom over the period 1991–1994 [76]; Unfortunately, other studies [77,78] showed that reduction of assay sensitivity with age was an ongoing issue. In a later study [79], 197 paired sera and oral fluid samples from infants, children and adults were tested using a class G capture amplification-based enzyme-linked immunosorbent assay (GACELISA) and the aforementioned GACRIA. The GACELISA performed better than the GACRIA; however, with adult samples sensitivity was only 60.8%. The issue of declining assay sensitivity with age when using class G antibody capture immunoassays lacks adequate explanation and remains to be resolved although application of enhanced modeling techniques may help alleviate the problem [80]. The possibility of using a completely different assay design for measuring rubella IgG in oral fluid samples has been explored by Ben Salah and colleagues [81]. In their study an indirect enzyme immunoassay format (Behring ELISA) was compared with GACELISA for detecting rubella IgG in an age stratified population. Using an optimized cut-off the sensitivity of the Behring ELISA was 89.8% and specificity was 92.0% compared to GACELISA optimised sensitivity and specificity of 92.4% and 93.2% respectively. Further studies are needed to confirm the utility of an indirect EIA format for rubella IgG detection in oral fluids and it will be seen in Section 3.3 that time-resolved fluorescence immunoassay can offer the sensitivity required. A rubella IgG time-resolved fluorescence immunoassay has been described [82] with a lower limit of detection of 0.2 IU/mL in sera and initial results on application to oral fluid appear promising.

The role of oral fluid assays for rubella IgM detection is more established [72,83] and these assays have found application in rubella surveillance and control. In rubella low incidence settings the potential for rubella misdiagnosis is high [84,85] and this is why, in the United Kingdom, a rubella oral fluid testing program has run since 1994 [86]. During January 1995–July 2003, 17,042 oral fluid samples from cases of clinically suspected rubella were tested as part of the program and the rate of case confirmation was 51% and the specificity was 55% [87]. The performance of oral fluid tests for rubella IgM detection is summarized in Table 2.

**Table 2:** Reported performance of rubella class IgM capture radioimmunoassays (MACRIA) and enzyme immunoassays (MACEIA).

Assay	Population Tested	Sensitivity	Specificity
Rubella MACRIA	Paired sera and oral fluids from 50 clinically diagnosed and serologically confirmed rubella cases and 91 paired sera and oral fluids from blood donors [28]	100% 1 day–5 weeks post onset	100%
Rubella MACRIA	Paired sera and oral fluids from 177 cases of notified rubella: 53 confirmed IgM positive by serology of paired serum and 124 confirmed IgM negative by serology [78]	81%	99%
Rubella MACRIA	Paired sera and oral fluids from 45 clinically diagnosed and serologically confirmed rubella cases and 149 paired sera and oral fluids from individuals with other non-rubella recent rash diseases [88]	84.4%	96%
Rubella MACEIA (Commercial)	Paired sera and oral fluids from 55 rubella cases from outbreak in Turkey and 111 paired sera and oral fluids from suspected congenital rubella syndrome cases in India [89]	>95%	>95%

Oral fluids, if collected soon enough following onset of illness and appropriately stored prior to testing are a valuable source of rubella virus genome which can be amplified by reverse transcription polymerase chain reaction (RT-PCR) and used to complement the results of antibody testing or sequenced so that the spread of virus can be tracked [31,90,91]. In a definitive study [92], Abernathy and colleagues showed that rubella RT-PCR testing of oral fluid confirmed more rubella cases than IgM testing of either serum or oral fluid samples collected in the first two days following rash onset and that the maximum number of confirmations of rubella cases was obtained by combining rubella RT-PCR and serology testing. Likewise, the value of virological surveillance for rubella using rubella RT-PCR to amplify viral genome in samples including oral fluids has been reviewed by Rota and colleagues [93].

## 3. Varicella: varicella vaccination and varicella oral fluid testing

### 3.1. Varicella

Primary infection with varicella zoster virus (VZV) manifests as varicella (chickenpox) and reactivation later in life produces herpes zoster (shingles). In the UK, traditionally, most cases of varicella occurred in 5–14 year olds; however, in recent years there has been a sharp increase in the prevalence of varicella in 1–4 year olds so that most cases, now, are reported in children aged 0–5 years [94,95]. Primary varicella infection in healthy children is generally a mild, self-limiting disease which typically presents as a cropping vesicular rash; however, in immunocompromised individuals and susceptible adults, particularly pregnant women, the infection can be more serious and even life threatening [96,97]. Finally, varicella infection during the first 20 weeks of pregnancy can result in miscarriage or foetal development anomalies (congenital varicella syndrome) while infection during the peripartum period can result in neonatal varicella which has a significant mortality rate [98].

### 3.2. Varicella Vaccination

An effective vaccine against varicella has been available for a number of years and universal childhood vaccination is undertaken in a number of countries (e.g., USA, Germany, Japan) although not the UK [99]. The UK policy of not introducing universal varicella immunisation is, in part, motivated by concerns that such a measure may shift the burden of primary disease to susceptible adults and increase shingles reactivations in later life due to a reduction in natural boosting in previously exposed individuals [100]. Targeted varicella immunisation strategies, instead of universal varicella vaccination of children, may have potential for preventing severe primary infections among adults. In the UK, selective varicella vaccination of susceptible adolescents is under active consideration [101]. An essential component for modelling the potential effectiveness of targeted vaccination and for monitoring trends in disease epidemiology is the capacity to accurately estimate the extent of VZV infection at a population level. Two methodologies are chiefly used to generate such estimates—serosurvey of VZV immunity [102] and recall of a history of chickenpox [103].

### 3.3. Application of Oral Fluid Testing to Varicella Control

Detection of VZV IgG in serum, using appropriately validated methods (e.g., fluorescent antibody to membrane antigen immunofluorescence assay—FAMA), has been shown [104,105] to correlate with a history chickenpox or vOka vaccination. FAMA is not amenable for testing large numbers of sera and is highly subjective, so alternative methodologies such as quantitative, standardised, VZV time-resolved fluorescence immunoassay [106,107] have been developed. For population based studies of immunity to VZV the logistics required to collect blood and the invasive nature of collecting blood samples are prohibitive. In addition, sero-surveys using stored blood samples, particularly from children, have the potential for bias as such samples are often collected for highly specific medical investigations. The utility of recall of history of chickenpox has been evaluated in a number of settings [108,109]. A number of studies have shown that recall of history of chickenpox is highly associated with serological evidence of chickenpox [110,111] and that no history of chickenpox has low association with a lack of serological evidence of chickenpox [112,113].

Detection of VZV IgG using oral fluid samples removes the biases and limitations associated with blood sampling or the need to rely upon uncertain recall of no history of chickenpox. Such an approach can be used to identify susceptible individuals as part of targeted vaccination initiatives. In a study of adolescents [114] use of a time-resolved fluorescence immunoassay to detect VZV IgG in oral fluid samples showed that significant vaccine wastage might occur if reported absence of history of chickenpox was used to determine the need for varicella vaccination. Serological testing followed by immunization of certain population groups without a history of chickenpox may prove cost effective [115,116] and oral fluid sampling may find useful application in these circumstances.

## 4. Conclusions

This review shows that oral fluid sampling and analysis can be of significant value in supporting disease control programmes and monitoring the impact of interventions such as vaccination. The technology for undertaking oral fluid analysis is currently highly specialised and only available at a limited number of laboratories and although such testing is of great value the lack of availability of more readily accessible assays can be seen as a drawback to deriving the full benefits of collecting these samples [84]. There is little reason to doubt that at the current rate of technological development, if the desire is sufficient, there will be an expansion in the availability of oral fluid testing kits, such as is the case with HIV oral fluid testing in the USA; however, it remains to be determined if such devices can achieve acceptable diagnostic accuracies compared to testing of fingerprick blood samples or blood collected by venepuncture [117]. The main application of oral fluid testing is in those circumstances where blood sampling is deemed not necessary, or is undesirable, and when the documented sensitivity and specificity of the oral fluid assay methodology to be used is considered sufficient for the purpose intended.
